# Medical Volcanic Eruptions

**Published:** 1869-06

**Authors:** 


### Editorial Department.

### Medical Volcanic Eruptions.

There are certain pent-up forces which annually require to be "let off" in order to preserve the medical profession from working upon too high pressure. After the recent eruptions upon the subjects of medical education, college fees and elevation of the *status* of the profession have passed, we shall, no doubt, return to our usual efficiency and usefulness as earnest workers and thinkers in the most educated, honored and useful profession in the world. There are defects in our system of medical education; all systems of education are doubtless capable of great improvement, and consequently may be said to possess defects. The great body of so-called educated men, are poorly educated; but very few of the number, comparatively, ever attain to any great eminence or high state of perfection. Education does not consist alone in knowledge of language, mathematics or science. It cannot be said that a man is educated because he has passed the university curriculum and received its honors; many, alas! have done this, and are still quite uneducated. It may be no fault of our institutions of learning that the graduates as a body do not attain to much perfection of knowledge; if a few attain this, it is all that can be expected of poor human nature. Those who know it best, understand why it is that many are called, while but few are chosen to be bright and shining lights in the world.

We have noticed an editorial article in one of our exchanges with some surprise, since it contains views so wholly at variance with our most earnest convictions as to the true comparative merit of the medical profession in this country and Europe, and also the relative rank of the medical profession with the other professions. We do not propose to devote space to a discussion which can end in no good, but cannot withhold from our readers a reply by Prof. S. D. Gross of Philadelphia, which is moderate, truthful and convincing. We only desire to suggest to our worthy cotemporary that the only educated men are those who have learned to *think and reason for themselves*, and that no other profession can at all compare in its training in this respect with the medical profession. The legal mind, it is true, weighs evidence with eminent ability, the theological scholar often draws conclusions from assumed premises with wonderful ingenuity, but it is reserved for the physician to more thoroughly test his facts, or supposed facts; to weigh the grains of proof with more even and more carefully adjusted balance, to gather truth and reject error upon a more perfect scale than is done by any other.

This much for the medical profession of all countries, but read Dr. Gross for the medical profession of this country.

### AMERICAN vs. EUROPEAN MEDICAL SCIENCE.

LETTER FROM PROF. S. D. GROSS.

To the Editor of the Medical Record:

Dear Sir:—In the editorial of your journal of the 15th instant, entitled "American vs. European Medical Science," you indulge in a criticism upon the American profession, which, whether just or unjust, is, to say the least, exceedingly severe, if not ungenerous. The impression which the article will make upon our foreign brethren, both in Great Britain and on the continent, will be anything but flattering to our national pride, and will, if allowed to have its sway, tend to confirm the disparaging opinions expressed of us by Sir Dominic Corrigan and others at the meeting of the British Medical Association at Dublin, in August, 1867. I, for one, must enter my caveat against such sweeping assertions.

You say practitioners of medicine may be divided into three classes—surgeons, nurses and physicians—to the first of whom you generously concede the palm of superiority over European surgeons, while to the physicians you assign not only a low, but, in my judgment, a contemptible rank, without education, skill, or scientific attainments. The reason for this superiority, you think, is obvious. It has, to use your expression, grown out of the necessities of our pioneer life. "Therefore, as operative surgeons, as inventors of mechanical appliances, we lead both continents." American surgeons will hardly endorse this assertion. They will hardly admit that they are mere operators and inventors of mechanical appliances. They believe that they are entitled to higher praise, and to a more dignified position in the scale of science and talent, if not actual genius. If, as Chassaignac justly remarked, in the presence of my colleague, Prof. Pancoast and myself, America at this moment wields the surgical sceptre of the world, the expression must have been designed to convey something more than mere mechanical perfection. An operation is one thing, and a very great thing, when it is properly done; but it is a much greater thing to know when it ought to be done, or, in other words, what are the conditions of the part and system which render such a procedure necessary, and what are the circumstances which will enable a man to tolerate the interference. I have always had the greatest respect for a surgeon who, when the emergencies require it, can dexterously cut off a limb, excise a tumor, or ligate an artery; but that feeling would be vastly diminished if, after the operation was over, I should find, upon careful inquiry, that the knife had been employed unnecessarily, or without that judgment and regard for life which every surgeon, worthy of the name, should possess, in order to fit him for a safe professional guide. I have always maintained—and I think in this opinion every rational person will coincide with me—that it is impossible for a man to be a surgeon, in the proper and more exalted sense of that term, unless he is a good physician, thoroughly familiar with the principles and practice of the healing art, considered in its widest bearing. The number of such practitioners in this country is legion. They are to be found everywhere, in the smaller towns and villages as in the larger cities, which are, perhaps, too generally supposed to contained all the science, wisdom, and skill of a nation; a conclusion certainly not borne out by the facts of the case either in the United States or in Europe.

It is said that the proof of the pudding is in the eating. If we compare the results of the practice and operations of American with those of European surgeons, it will be found that they are fully up to the general average, if, indeed, not far in advance. This statement is true alike of our civil and military experience. Where are there better, more enlightened, more able, skillful, or scientific surgical staffs than are to be daily seen in the wards of the hospitals of Philadelphia, New York, Boston, Baltimore, Richmond, Chicago, St. Louis, Cincinnati, Louisville, New Orleans, and other cities? Comparisons are odious. In my visits last summer to the hospitals of France, Italy, Austria, Prussia, Holland, Belgium, England, Scotland and Ireland, I saw nothing to induce the belief that the surgeons of those institutions, many of them veterans, whose names are as familiar to the American medical profession as household words, are, in any respect, superior to the same class of men among us, either in general or professional intelligence, in the art of diagnosis, in therapeutic refinement, or in manual dexterity. I take it for granted that human nature, civilized, refined and cultivated, is pretty much the same in all enlightened countries, and that professional superiority frequently exists more in the imagination than in the reality.

Are the invention and application of mechanical appliances mere mechanical affairs, requiring neither thought, talent, nor genius? Was the invention of printing, of steam navigation, of photography, and of a hundred other useful arts, a mere matter of chance, or was it the result of "brains," of genius, of the highest reasoning faculties of which human nature is capable? Let history answer these questions. Harvey discovered the circulation of the blood only after the most laborious observations and experiments, aided by the light of the inductive philosophy; and years elapsed before our own Sims was enabled to perfect his operation for the cure of one of the most loathsome affections incident to the female sex. There is a German adage, not over-refined, but so applicable to the present topic, that I can not refrain from quoting it: "A blind hog occasionally finds an acorn"—that is, chance sometimes reveals a great truth to a stupid fellow; but, generally speaking, a great truth can only be solved by great labor, talent and genius.

The late war—the unhappy war, as it is so justly styled—produced the best military surgeons the world ever saw; fertile in all kinds of resources, skilled in the application of remedies, thoroughly acquainted with the laws of hygiene, and most adroit in the use of the knife. They showed a familiarity with the most minute details of their profession, and a complete knowledge not only of "mechanical appliances," but of the higher principles of the healing art.

It is news to me, as it will be to many others, to learn as I do for the first time, that there is a class of practitioners on this side of the Atlantic called *nurses*. You say: "As nurses we are surely as good as the Europeans. We are all acquainted with practitioners of medicine who have little surgical ability, and no profound or philosophical attainment in science, who have no status in the profession, but who succeed in obtaining large and lucrative practice, and are successful with their cases, and are very popular with their patients." Wish to Heaven there were more of such practitioners. Though fools they may be, they are, according to your own showing, successful with their cases, and are very popular with their patients. Could learning and science accomplish more? Good nursing, as some one has justly said, is half the battle in the treatment of disease. If the other part be as well done, recovery will almost be certain.

"As *physicians*, we are just as certainly inferior to the Europeans." You seem to think that a medical practitioner cannot be good for anything unless he is a great scholar, equal, in point of attainments, to the leading scientific men of older countries, in England, France and Germany; and in support of this assertion you quote the language of Dr. John Brown of Edinburgh, the author of Rab and his Friends, of Horæ Subsecivæ, and other charming productions. "A physician is a man of universal acquirements." Your editorial says "a man may be a dexterous surgeon, or a kind and amiable nurse, without even knowing his alphabet, but no one can become a thorough, comprehensive physician without scholarship." Now all this sounds very well, and is equivalent to saying that a butcher may cut up an ox most dexterously without a knowledge of mathematics. A surgeon is no longer, as has been already stated, the mere servant of the physician, a mere operator, a mere mechanic, a position to which your language would reduce him; but he is an educated man, a thinking man, a man who uses his scalpel with brains, who knows what he is about, what is required of him to afford his patient the best chance for limb and life. "A physician is a man of universal acquirements." I was at Edinburgh last autumn, and I am sorry that the genial author of Rab and his Friends did not show me this extraordinary personage. He himself is a learned physician, and Sir James Y. Simpson, as we all know, abounds in varied knowledge. The Father of Medicine, nearly twenty-five centuries ago, made this pithy and oft-quoted remark: "Life is short, and art is long." Let us not forget this wise saying in an age when the physician has so much to learn.

"In our country we have, as a rule, been compelled to make thought, philosophy, science, attainments, culture, secondary to food, raiment, shelter, in the perpetual battle of existence." I do not see that this rule, of which you here speak, is peculiar to the United States, or to any one of the learned professions. The medical students of Europe are, as a rule, as poor as our own, if, indeed, not more so; and they have, therefore, just as much incentive, when they obtain their degree, to exert themselves for food, raiment and shelter. It is with them, as with us, with rare exceptions, a perpetual battle for existence. They have no more leisure than we have; they have no more weaith; they have no more brains; they have, as a bcdy, little, if any, more scholarship, thought, philosophy, science, attainment or culture. The American mind is the most active mind in the world; and, if the American physician is not as highly educated as the English, French and German—if he does not know as much Greek and Latin, mathematics, astronomy, and the more abstruse branches of a university education—he may justly pride himself upon his superiority of common sense, of Yankee ingenuity and smartness, seldom found in the same degree in any class of citizens in the old world. This Yankee shrewdness is a necessary outgrowth of the genius of our free institutions, where every one thinks and acts for himself, and where every boy, before he is fifteen years of age, is essentially a "live" man. Our partridges often run about with a part of the shell upon their backs, and our children often kick lustily before they have fairly escaped from the secundines of the mother. In Europe, especially on the continent, every one swears by the sleeve of his master; and the consequenee is that the mind of the pupil is still in a state of bondage long after he leaves the university.

But is it, as you assert, a fact that great scholarship is essential to the formation of a great physician? What constitutes a great physician? Is it a knowledge of the ancient and modern languages? or is it a knowledge of anatomy, physiology, pathology, diagnosis, materia medica and therapeutics? Which would a man, struck down by disease or accident, rather have, if he could have his choice—the great medical scholar, or the great physician, destitute of ancient lore, but thoroughly versed in the mysteries of the healing art, with all the resources of a fertile mind, quick to perceive the varying changes in his patient's condition, and ever ready to interpose the necessary remedies? It is not difficult to perceive upon whom the choice would fall.

I do not wish by the foregoing remarks to disparage learning in the physician. By no means. At the Teachers' Convention at Cincinnati, in 1867, no one went farther in this particular than myself; and I hope that the day is not distant—I wish to God it would be here to-morrow—when our medical schools will wake up upon this subject, and care more for the welfare of their pupils than the welfare of their pockets. All I desire is to show that great scholarship is not essential to the formation of a great physician; or, in other and more comprehensive terms, that a man may be a most accomplished practitioner of medicine, eminently skilled in all that pertains to his pursuit, and yet be a very poor general scholar, unable to conjugate a Greek or Latin verb, or to construe correctly a Greek or Latin sentence. If the medical profession of the present day is not as learned as it was in former times, it is far more scientific and far more able to cope successfully with disease. Medicine is a progressive science, and such have been its strides within the last quarter of a century that it takes all the time which a man can snatch from his daily toil to keep himself on a level with it. Just in proportion as he wanders from his profession does he slight its requirements and its solemn duties.

"In all our American colleges medicine has ever been, and is now, the most despised of all the professions which liberally-educated men are expected to enter. A few years ago an instructor in a leading university said to two young students, who were commencing their labors as medical students: 'Don't study medicine; anybody can be a doctor. Study law or theology.' I should be sorry to think that this statement embodied a general truth. My conviction is that the professors of our literary colleges have a high respect for the talents and learning of the medical profession. In no country in the world do physicians occupy so elevated a social position as in the United States. So far as my experience extends, this feeling is universal, not peculiar to any particular section, town, or city. In refinement, in general culture, in force of intellect, and in scientific attainment they have not, I am bold to say it, their superiors in either of the other professions; while in benevolence, in charitable acts, and in scientific research they are far in advance of them.

"Study law or theology," says this sage instructor. And what, if you do? Are lawyers and divines, as a body, more refined, learned, moral, or wise than medical men? If they are, it would require a long search, even with the aid of a much larger lantern than Diogenes employed to find a man, to furnish the proof. There are, and always must be, uneducated, coarse, ignorant men in every profession, the clerical not excepted. It could not from the very nature of things be otherwise; and what is true of this country is true, to a greater or less extent, of every country under heaven. 'Anybody can be a doctor!' Nothing is more true; but it is also true that anybody can be a lawyer or a preacher.

"The profession in America has been inclined to discourage rather than encourage original thought amongst its members." I agree with you, perfectly, Mr. Editor, in this opinion. It is, unfortunately, too much the custom of our physicians to depreciate each other, and yet there are many, many noble exceptions. American writers—young ones in particular—would rather, it seems to me, at any time—perhaps merely to display their *scholarship*—quote a foreign than an American book, although the former should be far the inferior in all the requisites of a great work. With all our independence and liberalized notions, we are still, in a very striking degree, toadies. The time has come when, as a great profession, we should do justice to ourselves, by encouraging one another in everything that is good and useful, cultivating good feeling, improving our system of medical education, rousing our schools to a stern sense of their duty, and fostering and improving our medical literature. Jealousy should be banished from among us; and every man, however humble or deficient in the scholarship of which you so much complain, should endeavor to do all he can to advance the honor and glory of his profession.

You seem to think that our delegates at the recent International Congress at Paris fully deserved the "snubbery" which they received—not as individuals, but as members of our profession—from their French brethren. My impression, from all I have seen of the Parisian physicians and surgeons, is that they intended no such slight. The Congress was essentially a French affair from beginning to end, intended to enable a few Frenchmen to ventilate themselves, as, I am told, they did, to the great disgust of many of the delegates of other countries. Paris is the hub of the universe, and consequently all outsiders are barbarians. The French are the most polite impolite people in the wide, wide world. They know nothing of our language, and are as ignorant of our profession as the Sioux Indians. Our English brethren, who know us much better, fully appreciate us, and speak in the warmest praise of our wonderful progress and elevated position.

Like yourself, I sigh for the coming of a golden age for our honored and beloved profession. I may not witness its advent, but assuredly the time will come when America will yield the medical sceptre of the world as she now does, in the opinion of Chassaignac, yourself, and many others, that of surgery. Hundreds of young men, good and true, thoroughly educated, eminently scientific, abounding in industry, fidelity and ambition, are, like so many bees, busy at work in every town and city in this great country, collecting honey from every flower, and throwing it into the great storehouse of the profession. Never was there such industry, such talent, such genius, such patient labor as there is at the present moment among the medical men of the Western World. I almost wish myself young again, that I might engage in the great race of glory and renown that are sure to be the lot of the young physicians and surgeons of this prolific, this illustrious age.

I have no desire to criticise your remarks respecting Philadelphia, in one of the closing paragraphs of your editorial. I only wish that they had never been written. So far as my information extends, I am right when I assert that the very best understanding has always existed between the medical men of Philadelphia and those of New York; and I am, therefore, sorry that you have indulged in expressions which cannot fail to wound the feelings of a large number of the physicians of the "City of Brotherly Love."

I have the honor to be, dear sir, very truly and respectfully, your friend and obedient servant, S. D. GROSS.

*Philadelphia, May* 20, 1869.

